# Genome-Wide Linkage in a Highly Consanguineous Pedigree Reveals Two Novel Loci on Chromosome 7 for Non-Syndromic Familial Premature Ovarian Failure

**DOI:** 10.1371/journal.pone.0033412

**Published:** 2012-03-13

**Authors:** Sandrine Caburet, Petra Zavadakova, Ziva Ben-Neriah, Kamal Bouhali, Aurélie Dipietromaria, Céline Charon, Céline Besse, Paul Laissue, Vered Chalifa-Caspi, Sophie Christin-Maitre, Daniel Vaiman, Giovanni Levi, Reiner A. Veitia, Marc Fellous

**Affiliations:** 1 Institut Jacques Monod, Université Denis Diderot, CNRS UMR7592, Paris, France; 2 Université Paris Diderot-Paris VII, Paris, France; 3 Department of Medical Genetics, University of Lausanne, Lausanne, Switzerland; 4 Department of Genetics, Hadassah University Hospital, Jerusalem, Israel; 5 Évolution des Régulations Endocriniennes, CNRS UMR7221, Muséum National d'Histoire Naturelle, Paris, France; 6 CEA/CNG, Institut de Génomique, Evry, France; 7 Institut Cochin, Université Paris Descartes, CNRS UMR 8104, Paris, France; 8 Inserm, U1016, Paris, France; 9 National Institute for Biotechnology in the Negev, Ben-Gurion University of the Negev, Beer-Sheva, Israel; 10 Inserm U933 Génétique de la Reproduction, Service d'Endocrinologie de la Reproduction, Hôpital Saint-Antoine, Université Pierre-et-Marie-Curie, Paris, France; Innsbruck Medical University, Austria

## Abstract

**Background:**

The human condition known as Premature Ovarian Failure (POF) is characterized by loss of ovarian function before the age of 40. A majority of POF cases are sporadic, but 10–15% are familial, suggesting a genetic origin of the disease. Although several causal mutations have been identified, the etiology of POF is still unknown for about 90% of the patients.

**Methodology/Principal Findings:**

We report a genome-wide linkage and homozygosity analysis in one large consanguineous Middle-Eastern POF-affected family presenting an autosomal recessive pattern of inheritance. We identified two regions with a LOD_max_ of 3.26 on chromosome 7p21.1-15.3 and 7q21.3-22.2, which are supported as candidate regions by homozygosity mapping. Sequencing of the coding exons and known regulatory sequences of three candidate genes (*DLX5, DLX6* and *DSS1*) included within the largest region did not reveal any causal mutations.

**Conclusions/Significance:**

We detect two novel POF-associated loci on human chromosome 7, opening the way to the identification of new genes involved in the control of ovarian development and function.

## Introduction

Premature ovarian failure (POF; MIM 311360 [OMIM]), clinically characterized by the early loss of normal ovarian function, is a cause of infertility in women under the age of 40 [1], [2], [3], [4]. POF can result from different ovarian defects, including early decrease in the primordial follicle pool, increased or accelerated follicular atresia and follicle growth blockade. The clinical characteristics of POF are a primary or secondary amenorrhea associated with elevated levels of circulating gonadotropins LH and FSH [5].

Although POF may be due to metabolic, autoimmune, infectious or iatrogenic causes, compelling evidence suggests that certain forms of the disease have a genetic etiology. Many POF cases are familial [6], [7], [8], [9] and specific genetic alterations or mutations have been associated with syndromic or non-syndromic forms of the disease. The most frequent anomalies are X chromosome alterations, including X chromosome monosomy (Turner syndrome) [10], X deletions and translocations, mutations of the *BMP15* gene [11], [12], [13], and premutations of the *FMR1* gene (Fragile X syndrome) [3]. POF-associated mutations were also identified in autosomal genes, such as *ATM* [14], [15], *FSHR* [16], *GDF9* [13], [17], *NOBOX* [18], [19], *AIRE* [20], *StAR* [21] and *FOXL2* [22], [23], [24], [25].

Despite these progresses, the etiology of POF remains unknown in about 90% of POF patients, suggesting that the causes of the disease are highly heterogeneous [5]. Therefore, a comprehensive identification of POF-predisposing genes is necessary to acquire a more accurate vision of the genetic causes of the disease.

Genome-wide linkage analysis has been successfully used to identify causal mutations in human diseases, including POF, for which such approach led to the identification of a 15.8 Mb region on chromosome 5 [7] and a mutation in *POF1B* on the X chromosome [6]. In order to identify novel POF-associated loci, we analyzed a large, highly consanguineous, non-syndromic POF family using a combination of genome-wide linkage and homozygosity mapping.

## Materials and Methods

### Ethical Statement

The research protocol was approved by the Medical Center Ethical Committee of the Hadassah University Hospital (Jerusalem). Written informed consent was obtained from all individuals included in the study.

### Participants and Data Collection

**Figure 1:**
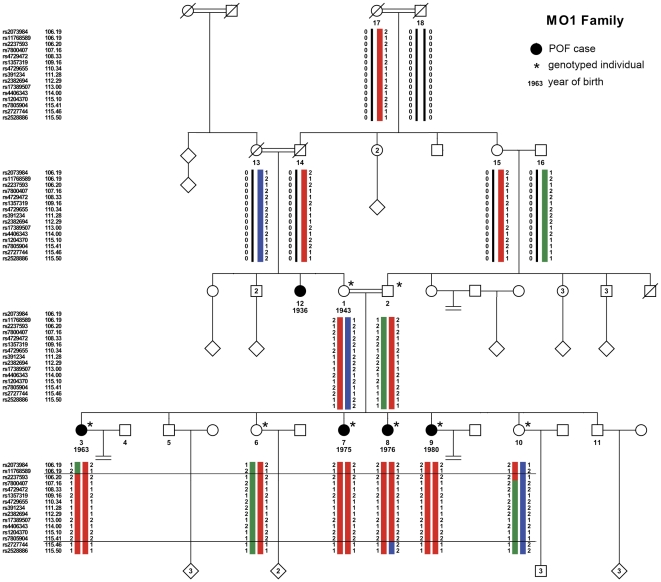
Pedigree of the consanguineous MO1 family, with haplotype reconstruction of the 7q21-22 region. Squares indicate males and circles indicate females. Black symbols indicate women affected with Premature Ovarian Failure. Year of birth, when known, is indicated below the number of the individual. Double connecting lines indicate consanguineous marriages, all known to be between first cousins even when the precise consanguinity loop could not be established. Asterisks mark the individuals included in the microsatellite and/or the SNP whole-genome genotyping studies. The haplotypes for the large 7q21-22 region were reconstructed with Merlin [26] for the individuals genotyped on the SNP array, and visualized with HaploPainter. For the sake of simplicity, the number of depicted SNPs was reduced, to 3 SNPs at the border of the shared homozygous block (2 SNPs out and 1 SNP in) and to approximately 1 informative SNP/cM inside the block. A red bar represents the ancestral disease haplotype transmitted both by the mother 1 and the father 2, and the green and blue bars represent the wild-type haplotypes. Recombination events in individuals 3 and 8 delimit the region of interest between the external markers rs11768589 and rs2727744 (horizontal lines).

A disease gene mapping was initiated in a large highly consanguineous Middle Eastern family (MO1). The MO1 Palestinian family (pedigree in Figure 1), with 5 affected women, was recruited through the Genetic Department of Hadassah Hospital in Jerusalem. Medical reports were obtained and completed when possible. Blood samples were collected for hormonal, genomic and cytogenetic studies. Karyotypes and genomic DNA isolation were performed by standard procedures.

The proband is the eldest of 6 sisters, 4 of them being affected with POF with primary amenorrhea. Their mother and father are first cousins, connected through at least 3 earlier consanguineous marriages. The non-affected mother and the affected maternal aunt were also born from a consanguineous marriage between first cousins, born from consanguineous marriages as well. The proband was diagnosed with POF at the age of 17 years because of amenorrhea, small and undeveloped breasts and streak gonads in ultrasound scan. The three other affected sisters were diagnosed with POF on the basis of a similar phenotype at ages between 18 and 20. All affected sisters presented with a normal uterus, but small ovaries with the appearance of “streak gonads” upon echography. Their height was within the normal range. Karyotype, *FMR1* premutation analysis and hormonal dosage were performed at the time of diagnosis (Table 1). No increased sedimentation rate or any other abnormal test for the presence of auto antibodies could be found in any patient. At the age of 19, the youngest POF patient suffered from a simultaneous bilateral ovarian cancer. After surgery, histological analysis revealed a gonadoblastoma on the right ovary, and a complex dysgerminoma/embryonic carcinoma/choriocarcinoma in the tumor of the left ovary. In addition to these four cases, a maternal aunt (individual 12, Figure 1) was diagnosed with POF and primary amenorrhea (her DNA was not available for the genome-wide linkage analysis). The non-affected sisters of the proband had menarche at ages within the normal range, and conceived naturally 2 and 3 children, respectively.

**Table 1:** Clinical and genetic data for the MO1 POF patients included in the study.

Individual	Type of amenorrhea	FSH (mIU/ml)	Estradiol (pg/ml)	LH (mUI/ml)	Karyotype	FMR1 premutation
MO1–3	primary	63.1	20.2	19.9	46,XX	No
MO1–7	primary	51.4	12.1	21.1	46,XX	No
MO1–8	primary	45.7	12.5	18.0	46,XX	No
MO1–9	primary	55.3	22.0	18.0	46,XX	No
**Reference values**		3–21	30–190	1.0–14.7		

### Microsatellites genotyping and SNP genotyping

Genotyping was performed on anonymous samples without knowledge of any personal identifiers at the Genotyping platform from the Institut de Génomique (Evry, France). After a genome-wide linkage analysis with 457 microsatellite markers (average autosomal marker spacing of 10 cM) that identified a significant linkage on chromosome 7, we performed a high-resolution genome-wide scan, using the Affymetrix GeneChip® Human Mapping 250 K Nsp Array (Affymetrix Inc., Santa Clara, CA). On this array, bi-allelic markers are equally distributed across the genome with a median physical distance between SNPs of 4.8 kb, an average distance of 11.2 kb, and an average 0.30 of heterozygosity. The Affymetrix genome-wide genotyping was performed according to the standard manufacturer's protocol (GeneChip® Mapping 250 K Assay Manual Rev. 3/96-Well Plate Protocol), using 250 ng of genomic DNA per sample. Washing and staining steps were performed with Affymetrix GeneChip® Fluidics Station 450 and scanning step with the Affymetrix GeneChip® Scanner 3000 7 G. Raw data were obtained with Affymetrix GeneChip® Operating Software (GCOS). Data were extracted with Affymetrix GeneChip® Genotyping Analysis Software (GTYPE) using BRLMM model as genotype calling method.

### Linkage analysis and Homozygosity mapping

After classical filtering of SNP markers for genotyping quality, mendelian errors and verification of Hardy-Weinberg equilibrium, multipoint parametric linkage analysis was performed with Merlin under the assumption of recessive inheritance, with a disease allele frequency of 0.0001 and a penetrance of 0.99 [26]. The haplotypes in the identified 7q21-22 region were reconstructed with Merlin and presented graphically with HaploPainter v.1.043 [27]. The homozygous regions were confirmed and displayed using the dedicated software KinSNP (http://bioinfo.bgu.ac.il/bsu/software/KinSNP/) [28]. POF patients in the MO1 family were searched for runs of shared homozygosity (ROSHs) with a minimum length of 1 cM according to the deCode genetic map. The degree of tolerance for genotyping errors within ROSHs was adjusted such that heterozygous calls surrounded by 15 or more shared homozygous SNPs were ignored.

### Sequencing of candidate genes

Candidate genes were amplified by PCR from genomic DNA of one affected POF patient and one non-affected sister (details of primers and PCR conditions are provided in Document S1). PCR products were sequenced by standard Sanger sequencing.

## Results and Discussion

### Linkage analysis in a highly consanguineous POF family

**Figure 2:**
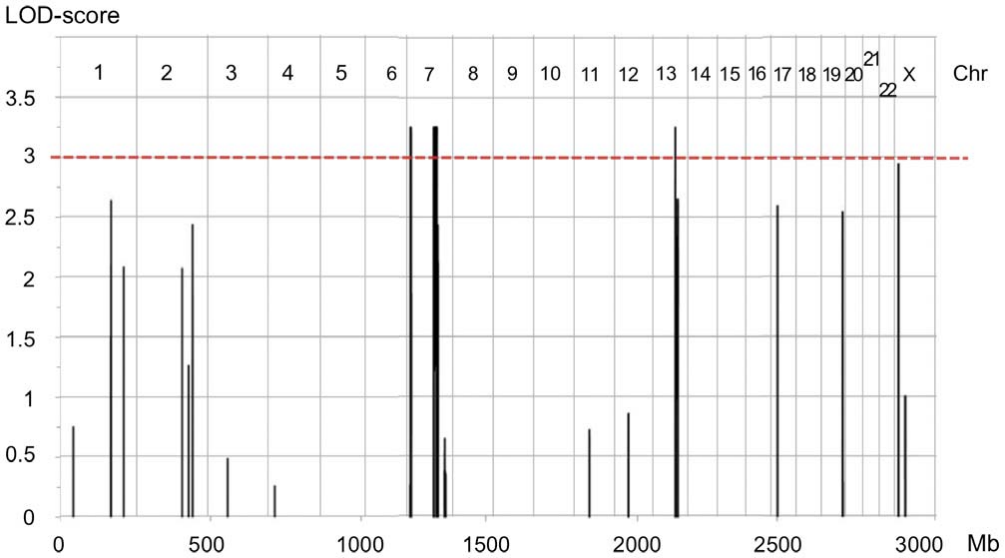
Whole genome linkage analysis for the MO1 family. The multipoint parametric LOD-score is plotted against the physical location of markers along the genome. The chromosome number is indicated in the upper part of the graph. Genotype analysis was performed on the Affymetrix GeneChip® Human Mapping 250 K *Nsp* Array (Affymetrix Inc., Santa Clara, CA). A significant LOD-score was obtained for two regions on chromosome 7. The apparent single peak on chromosome 13 encompasses two distinct very small peaks.

The highly consanguineous MO1 family (Figure 1) includes 5 POF patients in two generations (familial and clinical details in Material and Methods and Table 1). Although it was not possible to retrieve further familial information to assess earlier consanguinity loops, the high degree of consanguinity in this family strongly suggested an autosomal recessive inheritance of the disease allele from a common ancestor. A first genome-wide linkage analysis with 457 microsatellite markers identified a 10.5 Mb-long region within 7q21-22 (maximum LOD-score (LOD_max_) of 3.8). A second whole-genome scan with a 250 K SNP array identified four chromosomal regions with significant linkage under a recessive model (LOD_max_ = 3.26, Figure 2 and Table 2). It confirmed the significant linkage for a 9.96 Mb region on 7q21.3-22.2. It identified also an additional 2.56 Mb-long linkage region in 7p21.1, and two narrow segments, each spanning less than 0.2 Mb, within a short region of 6.6 Mb in 13q14. These regions had not been detected by the first genome-wide scan, since they were entirely framed by the analyzed microsatellites.

**Table 2:** Characteristics of significant LOD-score loci identified by genome-wide linkage in the MO1 family.

Chrom	7		7		13		13	
**Cytoband**	7p21.1	7p15.3	7q21.3	7q22.2	13q14.12		13q14.3	
**LOD max**	3.257		3.257		3.257		3.257	
**# of SNPs**	297		643		25		27	
**Flanking SNP**	rs13222101	rs7806550	rs11768589	rs17152355	rs4245330	rs2248414	rs536338	rs797493
**Position (cM)**	31.654	34.696	106.189	115.436	47.21	47.495	53.166	53.377
**Size (cM)**	3.042		9.247		0.285		0.211	
**Position (bp, in GRCh37)**	17723272	20278642	95615196	105575202	44661321	44798197	51103461	51261023
**Size (Mb)**	2.56		9.96		0.14		0.16	
**Homozyg.**	YES		YES		NO		NO	
**# of genes**	11+5 pseudogenes		166+39 pseudogenes		1 hypothetical		1 (2 terminal exons)	
**Candidate Genes**			SHFM1, DLX5, DLX6					

The flanking SNPs are the first ones outside the LOD_max_ peak, defined as having a LOD-score<LOD_max_-2 or LOD-score<LOD_max_-1. The Homozyg. line indicates whether the linkage peak was detected as a run of shared homozygosity by the KinSNP analysis.

Both regions on chromosome 7 are supported as candidate regions for the disease gene because they are detected as large runs of shared homozygosity by KinSNP (data not shown), and by a correct transmission of haplotypes, as reconstructed by Merlin. The four affected sisters shared a common disease haplotype, at the homozygous state because of the inheritance from a common ancestor through the paternal and maternal branches. Their non-affected sisters were either heterozygous (individual 6) or non-carrier (individual 10) for the disease haplotype (Figure 1). In addition, as the haplotypes confirmed that the affected sisters were not hemizygous for these regions, the mutation is not a large-scale deletion. Five larger ROSHs are detected by KinSNP, but in all these, the non-affected mothers and all the daughters shared the same haplotype at the homozygous state, which is incompatible with the transmission of the disease in the family. Therefore, the candidate region on the 7q21.3-22.2 is the largest homozygous stretch compatible with linkage.

The two peaks on chromosome 13 are not detected as ROSHs by KinSNP, due to the small size of the homozygous stretches. However, a visual inspection of the haplotypes in the KinSNP output confirmed that these peaks indeed contain a range of SNPs that are homozygous by descent in the four affected sisters, and heterozygous in the non-affected sisters and the non-affected mother (data not shown). Additional linkage analysis along 0.2, 0.4, 0.6, 0.8 and 1 cM grids on chromosome 13 showed that the positive peaks disappeared only with the setting of 1 cM. Thus, those peaks probably do not result from linkage disequilibrium and are likely to reflect identity-by-state homozygosity.

In addition, the SNP-based linkage analysis identified a near-significant region of 0.19 Mb on Xp22.33, that spans the boundary between the pseudo-autosomal region and the X specific region (LOD = 2.94, figure 2). This peak is detected as a ROSH by KinSNP. However, the analysis of the haplotypes revealed that the non-affected mother, the healthy sisters, as well as the affected daughters, shared the same haplotype at the homozygous state for 15 of the 20 SNPs within the peak (data not shown). Therefore, it is highly unlikely that this locus contains the mutation responsible for the POF phenotype in this family.

### Sequencing of POF candidate genes in the MO1 family

In total, the two loci on chromosome 7 span 12.29 cM and 12.52 Mb, and contain 177 genes (details of positions, sizes and genes content for all peaks with a LOD-score above 3 are presented in Table 2, and a list of all the genes located in these regions in Table S1).

We cannot exclude the smaller loci on chromosome 13 as possibly containing the mutation involved in this familial POF, however they do not contain any evident functional candidate gene, if any gene at all. The first locus on chromosome 13 could possibly contain one gene (C13orf44), but the corresponding RefSeq sequences were permanently suppressed from the Gene database because there is currently insufficient support for the transcript and the protein (GeneID 79024). The second locus on chromosome 13 contains only the 2 terminal exons of two transcripts of the DLEU1 gene, a long non-coding RNA gene with multiple splicing variants implicated as a tumor suppressor in B-cell chronic lymphocytic leukemia.

Similarly, the region on 7p21.1 does not contain any obvious functional candidate genes. *TWIST1* was formerly known as the BPES3 locus, because eyelid features of some patients with Saethre-Chotzen syndrome, due to mutations in *TWIST1*, were similar to those presented by patients with BPES. Nevertheless, this specific phenotype observed in those patients affected with Saethre-Chotzen syndrome was later recognized to be due to phenotypic variability. In addition, patients with Saethre-Chotzen syndrome do not present any ovarian defects. Therefore *TWIST1* cannot be considered as a POF candidate gene.

On the contrary, the region on 7q21.3-22.3 includes at least three POF functional candidate genes. *DLX5* and *DLX6* code for two closely linked homeobox transcription factors, involved in the control of steroidogenesis [29]. The allelic reduction of *Dlx5* and *Dlx6* in the mouse is associated with a POF-like phenotype, with an early reduction of fertility and a rapid and premature follicular depletion. This study also provided evidence supporting a reciprocal regulation between *Dlx5*, *Dlx6* and *Foxl2*, a transcription factor essential for ovarian development and function, also implicated in POF [30], [31], [32]. Another potential candidate gene is *SHFM1*, located in close proximity of the *DLX5/DLX6* locus. Its nematode ortholog, *dss-1*, is functionally conserved during evolution and was shown to be required for oogenesis and normal female fertility in *C. elegans* (with no impact on gonad development or on male fertility) [33]. The murine *Dss1* is expressed in the early genital tubercle during fetal development, and in the early genital bud of the newborn mice [34]. Although these three genes are implicated in the etiology of the Split Hand/Split Foot Malformation Type I syndrome (SHFM1, MIM%183600) [35], their possible function in the ovary prompted us to sequence them in the MO1 family. Sequencing of the coding, promoter and known enhancers regions of *DLX5* and *DLX6* did not show any DNA variants segregating with the disease. Likewise, we did not find any variants in the promoter and coding sequence of *SHFM1*.

### Conclusions

We have indentified two novel POF-associated loci on chromosome 7, that do not coincide with the regions identified by genome-wide linkage in the two previous studies of familial POF cases: the *POF1B* gene on the X chromosome, and a 15.8 Mb region in 5q14.1-q15 [6], [7]. In addition, these regions on chromosome 7 are not included and do not contain the CNVs that were detected as statistically different from controls in POF patients by Aboura and colleagues [36]. The identification of two other putative loci in our study highlights the important genetic heterogeneity of this disease.

The fact that the severe ovarian cancer affecting the youngest POF patient (individual 9) was bilateral and simultaneous strongly suggests a genetic origin. However, the association between POF and ovarian cancer is highly unusual and, to our knowledge, was never described before. Besides, a direct relation between POF and ovarian cancer is doubtful since only one POF-affected sister developed a cancer at a young age. Therefore it is more likely that the co-occurrence of these diseases in this patient could be related to the high familial consanguinity.

We could not find any sequence variant segregating with the disease in the coding and known regulatory sequences of *DLX5/6* and the coding sequence of *SHFM1*. Although we cannot exclude the existence of still unknown regulatory features, possibly affecting both *SHFM1* and *DLX5/DLX6*, which could harbor the causal mutation for POF in our family, the most likely hypothesis is that the mutation is present in the coding sequence of another gene located in the 7p or the 7q regions.

Among those genes, other possible candidates include *SMURF1*, the *CYP3* gene family and *VGF*. *SMURF1* encodes an E3 ubiquitin ligase specific of regulatory SMAD proteins, that was shown in rat and human granulosa cell lines to ubiquitinylate R-Smad 1 and 5, two of the regulatory Smads activated by oocyte-secreted BMP15, a known POF gene [37]. The *CYP3* gene cluster in 7q includes four genes, *CYP3A43*, *CYP3A4*, *CYP3A7* and *CYP3A5*, encoding cytochrome P450 enzymes, known to be implicated in drug metabolism and synthesis of cholesterol and steroids. Although some of the CYP3A genes appear to have a restricted expression in the liver and to be mainly responsible for drug detoxification, we cannot exclude a role in steroid synthesis in the ovary [38]. *VGF* encodes a 68-kDa precursor of multiple bioactive peptides with diverse neuroendocrine functions, expressed abundantly in the brain, and in peripheral endocrine tissues including the pituitary gland. In addition to a role in the regulation of energy homeostasis, *VGF* could also regulate reproduction, since homozygous Vgf-null mice are infertile, presenting a delayed sexual maturation, incomplete mammary development and ovaries with only primary and atretic follicles, apparently due to an abnormal pitituary gonadotropin content [39].

As the two loci identified on chromosome 7 contain too many genes for a direct sequencing approach in search of the causal mutation, we plan to proceed to an exome-sequencing in the MO1 family. Hopefully, this study will enable us to identify the mutation implicated in Premature Ovarian Failure in this family and might lead to the identification of one or several genes involved in the control of ovarian function and development.

## Supporting Information

Document S1 **Primers and conditions of PCR used for sequencing** ***SHFM1, DLX5*** **and** ***DLX6*** **.**
(DOC)

Table S1 **List of genes present in the loci identified on chromosomes 7 and 13 by genome-wide linkage in the MO1 POF family.** The lists were established using the NCBI Genome Viewer, using the rs number of the border SNPs, as given in Table 1. Pseudogenes are written in grey.
(XLS)
