# Supplementary material for: Genome-Wide Linkage in a Highly Consanguineous Pedigree Reveals Two Novel Loci on Chromosome 7 for Non-Syndromic Familial Premature Ovarian Failure
Source: PLoS One. 2012 Mar 13;7(3):e33412. doi: 10.1371/journal.pone.0033412 (PMC3302824; doi:10.1371/journal.pone.0033412)
Supplement: Document S1 — Primers and conditions of PCR used for sequencing SHFM1, DLX5 and DLX6 . (DOC) [file pone.0033412.s001.doc]

**Document S1.**

**PCR conditions and primers for amplification of human *DLX5* and *DLX6*, promoters, enhancers and regulatory regions, and *SHFM1*.**

**Sequencing was performed with internal primers.**

**Human DLX5**

**PCR primers:**

Fragment 1: Promoter

prHuDLX5F1-1F: AATGGCAGTGTGTATCAGGTGACT

prHuDLX5F1-1R: CCTGCTGCAAAGACATCCTACTTA

**PCR conditions: (with DMSO)**

1/ 95°C 10 mn

2/ 95°C 40 s

3/ 59°C 30 s

4/ 72°C 1 mn

5/ Go to 2 34 x

6/ 72°C 10 mn

Fragment 2: Promoter

prHuDLX5F2-1F: ATGGGGATTGGGAGAGACTTTTAT

prHuDLX5F2-1R: GTTCCAATCAGAAGCCTCAAAAG

**PCR conditions: (with DMSO)**

1/ 95°C 10 mn

2/ 95°C 40 s

3/ 60°C 30 s

4/ 72°C 1 mn

5/ Go to 2 34 x

6/ 72°C 10 mn

Fragment 3: promoter + exon 1

prHuDLX5F3-1F: CTTCCTTCTTGCCTCCATCCTAC

HuDLX5Ex1-3R: gctctggtctaagcagacatgg

Fragment 4: Exon 1

HuDLX5Ex1-1F: ACAGAGACTTCACGACTCCCAGT

HuDLX5Ex1-1R: GTCTGAGTCCTACTCCCTTCTGC

Fragment 5: Exon 2

HuDLX5Ex2-1F: ATTAATCAGAGGCTATCCCGCTTT

HuDLX5Ex2-1R: AGCTCAGGCAGGTCTAGTGCAT

Fragment 6: Exon 3

HuDLX5Ex3-1F: ACTCAGGTGAAAATCTGGTTTCca

HuDLX5Ex3-1R: ATAGAGCCCCGACTTCTGTTTTCT

**PCR conditions:**

1/ 95°C 10 mn

2/ 95°C 40 s

3/ 62°C 30 s

4/ 72°C 40 s

5/ Go to 2 34 x

6/ 72°C 10 mn

**Human DLX6**

**PCR primers:**

Fragment 1: Promoter

**prHuDLX6-2000F:** ACCGAGAGGATCTTCAGGTCT

**prHuDLX6-1128R:** CAGAAGCTACTGTAATGCTTTCAA

Fragment 2: Promoter

**prHuDLX6-1267F:** TCAATTGGAATTGTAATCGAGAGA

**prHuDLX6-346R:** TAAGGCAGAGATTTTAAGGTGGAT

**PCR conditions:**

1/ 95°C 10 mn

2/ 95°C 40 s

3/ 62°C 30 s

4/ 72°C 1 mn

5/ Go to 2 34 x

6/ 72°C 10 mn

Fragment 3: Exons 1 & 2

**HuDLX6-Ex1-2F:** CTTCCTCCTCCCTTTGAGTTAACAAG

**HuDLX6-Ex1-2R:** cccgGGACTCAGCCCGAACTGC

**PCR conditions: (with DMSO)**

1/ 95°C 10 mn

2/ 95°C 40 s

3/ 67°C 30 s

4/ 72°C 1 mn 10 s

5/ Go to 2 34 x

6/ 72°C 10 mn

Fragment 4: Exon 3

**HuDLX6-Ex3-F1:** CACACGGGCAGAGTGGAC

**HuDLX6-Ex3-R1:** CTTCCATCCTTGGTTGACTAGG

**PCR conditions:**

1/ 95°C 10 mn

2/ 95°C 40 s

3/ 59°C 30 s

4/ 72°C 35 s

5/ Go to 2 34 x

6/ 72°C 10 mn

Fragment 5: Exon 4

**HuDLX6-Ex4-F1:** CAATAACTTTAGGTATCCAGGCAAG

**HuDLX6-Ex4-R2:** GAAGGGAAGTGAGGGAGGAG

**PCR conditions: (with DMSO)**

1/ 95°C 10 mn

2/ 95°C 40 s

3/ 62°C 30 s

4/ 72°C 1 mn 40 s

5/ Go to 2 34 x

6/ 72°C 10 mn

**Regulatory regions between SHFM1 and DLX6**

REG1: 5’ of SHFM1

Fragment 1

**Hu5’dssRG1-F1:** GCCATGTATGGTTGCGACTTAAT

**Hu5’dssRG1-R1:** GACAAGTTTACCACATGATGGAC

Fragment 2

**Hu5’dssRG1-F2:** CCTGAAGCCTGATCAGAATTA

**Hu5’dssRG1-R1:** GCCACACATCTGTGAGCAAGTAT

**PCR conditions: (with DMSO)**

1/ 95°C 10 mn

2/ 95°C 40 s

3/ 60°C 30 s

4/ 72°C 1 mn

5/ Go to 2 34 x

6/ 72°C 10 mn

REG2:

[Human](http://www.ensembl.org/Homo_sapiens/Info/Index) [GRCh37]: [217743 bp at 3' side: distal-less homeobox 6](http://www.ncbi.nlm.nih.gov.gate1.inist.fr/entrez/viewer.fcgi?val=224514692&db=Nucleotide&from=34668133&to=34672202&view=gbwithparts&RID=W8RT9YPC016)

**Hu5’Dlx5-6RG2-F1:**TTCATCTGCTGAAGAAAAGATGC

**Hu5’Dlx5-6RG2-R1:** TGGTATCTCAGGCACATCTCCAT

**Hu5’Dlx5-6RG2-F2**: GGGGCTTACACTACAGTTTGCC

**Hu5’Dlx5-6RG2-R2:** GTGGGGGATTATCTGGAGACTGG

**PCR conditions:**

1/ 95°C 10 mn

2/ 95°C 40 s

3/ 61°C 30 s

4/ 72°C 50 s

5/ Go to 2 34 x

6/ 72°C 10 mn

REG3:

[Human](http://www.ensembl.org/Homo_sapiens/Info/Index) [GRCh37]: [171367 bp at 3' side: distal-less homeobox 6](http://www.ncbi.nlm.nih.gov.gate1.inist.fr/entrez/viewer.fcgi?val=224514692&db=Nucleotide&from=34668133&to=34672202&view=gbwithparts&RID=W8SPPSEB016)

**Hu5’Dlx5-6RG3-F1:** TTCCAGTTAAGTCATGTTAGGACAA

**Hu5’Dlx5-6RG3-R1:**CCTCGTTTTTAAAGCAAAGAACC

**Hu5’Dlx5-6RG3-F2:** GTAAATAGACATCTTAGAAAAC

**Hu5’Dlx5-6RG3-R2:** GACACAGTTTAACCAATACG

**Hu5’Dlx5-6RG3-F3:** GTTATATACCAAACAGACATGC

**PCR conditions: (with DMSO)**

1/ 95°C 10 mn

2/ 95°C 40 s

3/ 62°C 30 s

4/ 72°C 1 mn 20 s

5/ Go to 2 34 x

6/ 72°C 10 mn

REG4:

[Human](http://www.ensembl.org/Homo_sapiens/Info/Index) [GRCh37]: [13226 bp at 3' side: distal-less homeobox 6](http://www.ncbi.nlm.nih.gov.gate1.inist.fr/entrez/viewer.fcgi?val=224514692&db=Nucleotide&from=34668133&to=34672202&view=gbwithparts&RID=W8SY34V5013)

**Hu5’Dlx5-6RG4-F1:** GCATCAAAATAAATTGTGCGTTG

**Hu5’Dlx5-6RG4-R1:** CCTTAACCAGGAGAAGGGAGAGA

**Hu5’Dlx5-6RG4-F2:** CAAGGATAAATGGCATCGAAC

**Hu5’Dlx5-6RG4-R2:** CAATCATGGGCTCCTTGGGCTG

**PCR conditions: (with DMSO)**

1/ 95°C 10 mn

2/ 95°C 40 s

3/ 60°C 30 s

4/ 72°C 40 s

5/ Go to 2 34 x

6/ 72°C 10 mn

**Human SHFM1:**

The promoter was analyzed as regulatory region 1 (see above)

Fragment 1.

**HuDSSf1/1F:** gagaatgggaaagacatgatgaa

**HuDSSf1/1R:** gctcaaagaactgcaaagagtgt

**PCR conditions:**

1/ 95°C 10 mn

2/ 95°C 40 s

3/ 60°C 30 s

4/ 72°C 40 s

5/ Go to 2 34 x

6/ 72°C 10 mn

Fragment 2

**HuDSSf1/2F:**gcattacatgactagcacagtgg

**HuDSSf1/2R:** cagaacataaaaggacatggaaaa

**PCR conditions:**

1/ 95°C 10 mn

2/ 95°C 40 s

3/ 61°C 30 s

4/ 72°C 50 s

5/ Go to 2 34 x

6/ 72°C 10 mn

**Enhancer regions between human DLX5 and DLX6**

Enhancer eI56i:

**HuI56iF1:** TTCGCATTGTTACATTAGGAAAAA

**HuI56iR1:** TGTTATGCACTCACAGTGGTTTG

**PCR conditions:**

1/ 95°C 12 mn

2/ 95°C 40 s

3/ 60°C 40 s

4/ 72°C 50 s

5/ Go to 2 34 x

6/ 72°C 10 mn

Enhancer eI56ii:

**HuI56iiF1:** TATTCTTGGTTGAGTGAGCACAT

**HuI56iiR1:** TTGCTATTTTCTTAATTCTCGCTCT

**PCR conditions: (with DMSO)**

1/ 95°C 12 mn

2/ 95°C 40 s

3/ 61°C 40 s

4/ 72°C 40 s

5/ Go to 2 34 x

6/ 72°C 10 mn
